# Supplementary material for: Long-Term Prognosis of Patients with Myocardial Infarction Type 1 and Type 2 with and without Involvement of Coronary Vasospasm
Source: J Clin Med. 2020 Jun 2;9(6):1686. doi: 10.3390/jcm9061686 (PMC7356040; doi:10.3390/jcm9061686)
Supplement: Supplementary file 1 [file jcm-09-01686-s001.pdf]

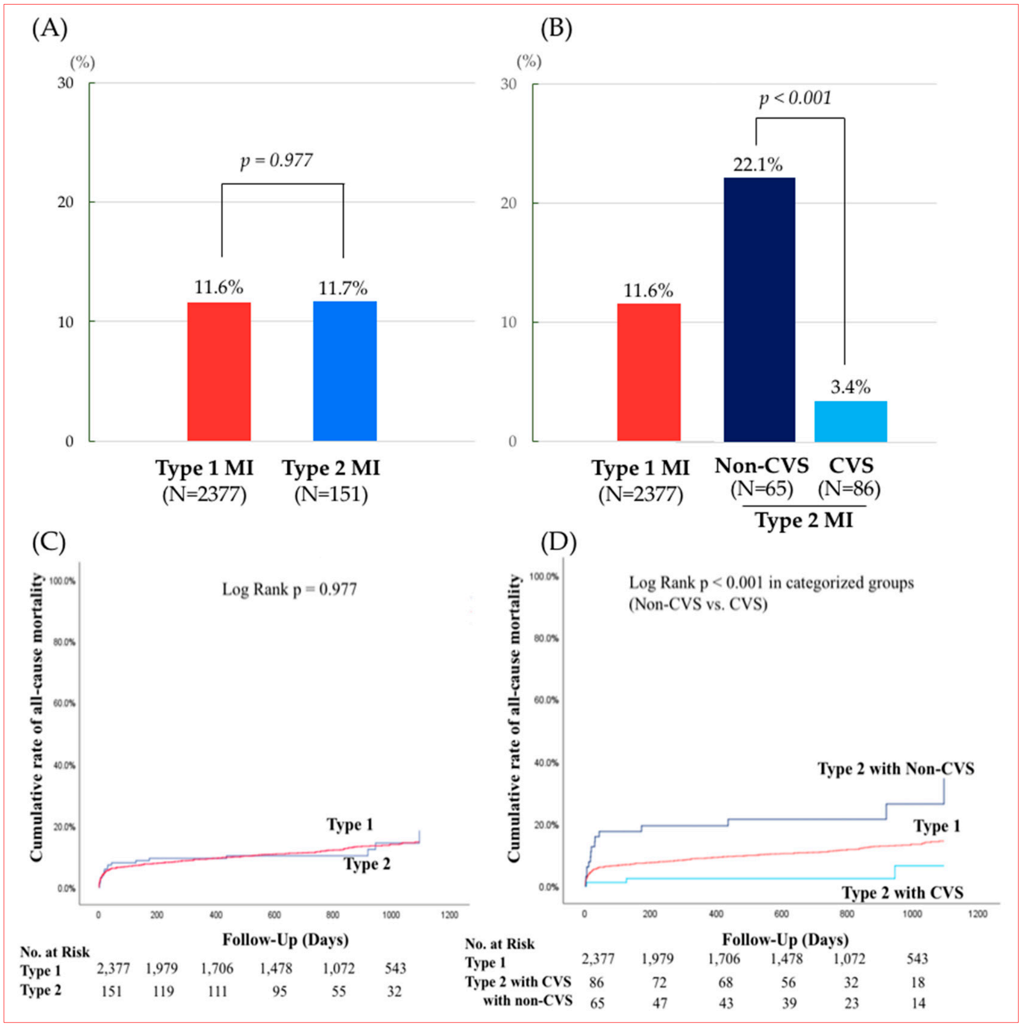

Supplemental Figure. All-cause mortality and Kapan-Meier estimates with excluding patients diagnosed by CPK alone. (Upper) All-cause mortality between Type 1 MI and Type 2 MI (A) and the mortality among the 3 groups, when Type 2 MI is stratified into 2 groups (B) over a 3-year period. (Lower) Kaplan-Meier estimates of all-cause mortality between Type 1 MI and Type 2 MI (C) and the mortality among the three groups, when Type 2 MI is stratified into two groups (D) over a 3-year period. Abbreviations are shown in Figure 2 and 3.
